# The battle between the innate immune cGAS-STING signaling pathway and human herpesvirus infection

**DOI:** 10.3389/fimmu.2023.1235590

**Published:** 2023-08-02

**Authors:** Ximing Jin, Wenjia Wang, Xinwei Zhao, Wenhua Jiang, Qingqing Shao, Zhuo Chen, Cong Huang

**Affiliations:** ^1^ Institute of Integrated Traditional Chinese and Western Medicine, Tongji Hospital, Tongji Medical College, Huazhong University of Science and Technology, Wuhan, China; ^2^ Department of Integrated Traditional Chinese and Western Medicine, Tongji Hospital, Tongji Medical College, Huazhong University of Science and Technology, Wuhan, China

**Keywords:** cGAS, STING, human herpesvirus, innate immunity, immune escape

## Abstract

The incidence of human herpesvirus (HHVs) is gradually increasing and has affected a wide range of population. HHVs can result in serious consequences such as tumors, neonatal malformations, sexually transmitted diseases, as well as pose an immense threat to the human health. The cGAS-STING pathway is one of the innate immune pattern-recognition receptors discovered recently. This article discusses the role of the cGAS-STING pathway in human diseases, especially in human herpesvirus infections, as well as highlights how these viruses act on this pathway to evade the host immunity. Moreover, the author provides a comprehensive overview of modulators of the cGAS-STING pathway. By focusing on the small molecule compounds based on the cGAS-STING pathway, novel targets and concepts have been proposed for the development of antiviral drugs and vaccines, while also providing a reference for the investigation of disease models related to the cGAS-STING pathway. HHV is a double-stranded DNA virus that can trigger the activation of intracellular DNA sensor cGAS, after which the host cells initiate a cascade of reactions that culminate in the secretion of type I interferon to restrict the viral replication. Meanwhile, the viral protein can interact with various molecules in the cGAS-STING pathway. Viruses can evade immune surveillance and maintain their replication by inhibiting the enzyme activity of cGAS and reducing the phosphorylation levels of STING, TBK1 and IRF3 and suppressing the interferon gene activation. Activators and inhibitors of the cGAS-STING pathway have yielded numerous promising research findings *in vitro* and *in vivo* pertaining to cGAS/STING-related disease models. However, there remains a dearth of small molecule modulators that have been successfully translated into clinical applications, which serves as a hurdle to be overcome in the future.

## 1 Introduction

From ancient times to the present, the interaction between virus and host has never stopped. In the face of a viral invasion, the human immune system has evolved so that it can defend the human body against several diseases; nevertheless, in response to the strong immune defense system, viruses have also ingeniously evolved several strategies to evade immune surveillance and antiviral immune responses. There are nine types of human herpesvirus (HHV), these viruses can cause different diseases, including genital herpes, neonatal encephalitis, varicella, herpes zoster, roseola infantum, Kaposi’s sarcoma, infectious mononucleosis, and Hodgkin’s lymphoma. HHV can affect individuals of all age groups, from newborns to the elderly. Furthermore, it can cause serious consequences such as cancer or birth defects. HHV has already led to serious health problems worldwide.

At present, the primary antiviral drugs are nucleoside analogs that mainly comprise acyclovir and glucocorticoids. However, in clinical settings, no effective vaccines are available for many types of HHV (1). Therefore, there is an urgent need to discover new antiviral targets and develop new antiviral strategies.

The body’s first line of defense against pathogen invasion is the natural immune system. There is no doubt that it plays a crucial role in antiviral immunity. Many pattern recognition receptors (PRRs) are important components of this barrier; they recognize pathogen-associated molecular patterns and activate a sequence of signaling pathways in the body to generate a natural immune response.

Cyclic GMP – AMP synthase (cGAS) is a newly discovered intracellular nucleic acid receptor (2). It can detect the host’s double-stranded DNA produced as a result of body damage or foreign double-stranded DNA. Recent studies have reported that cGAS can detect genetic material produced by bacteria, thereby initiating a chain of immune responses.

Stimulator of interferon genes (STING) is a molecule that connected to cGAS downstream. Many studies have reported the role of the cGAS-STING pathway in various systemic diseases. The cGAS-STING pathway plays a role in the immune responses to various conditions, including respiratory system diseases, circulatory system diseases, digestive system diseases, nervous system diseases, viral infection, autoimmune diseases, tumorigenesis, and aging.

In the beginning, most researchers either focused on the interaction between one kind of virus or one disease and the cGAS-STING pathway or on the regulatory effects of a molecule on the cGAS-STING pathway. Besides, the cGAS-STING pathway is rarely involved in the immune escape of viruses.

In present study, we emphasize the role of the cGAS-STING pathway in various systemic diseases by briefly summarizing its role in diseases occurrence, and emphatically discussing the research progress on the cGAS-STING pathway in anti-human herpesvirus infections. Furthermore, we present some regulatory molecules for cGAS and STING. Our study provides new ideas for identifying more effective and comprehensive antiviral targets and developing new antiviral drugs and vaccines. It also provides a research basis for discovering effective therapeutic drugs for diseases associated with the cGAS-STING pathway in clinical settings.

## 2 cGAS-STING pathway

**Figure 1:**
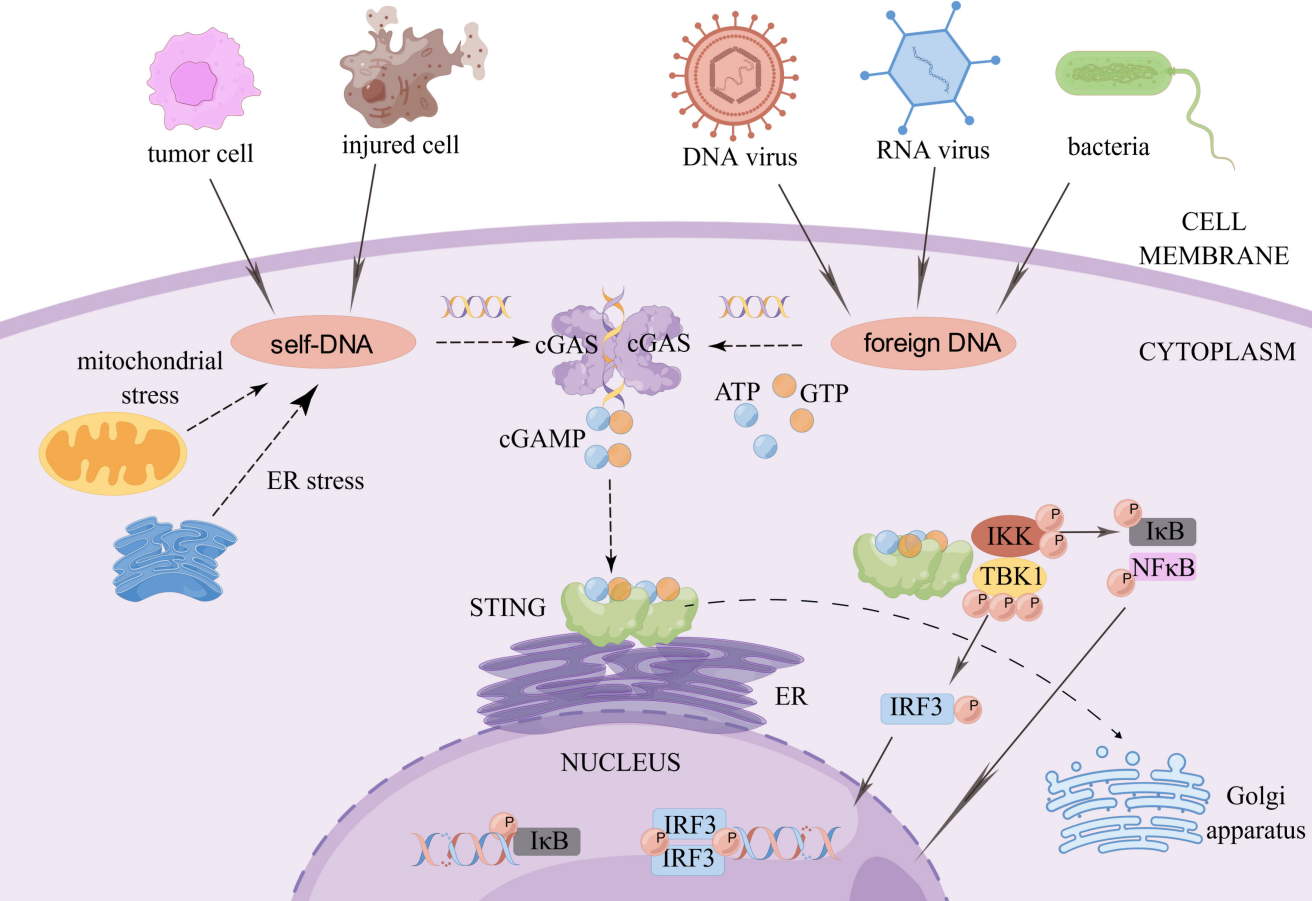
The cGAS-STING signaling pathway. When exogenous pathogen DNA invades, or when the organism experiences cellular damage resulting in leakage of self DNA, the intracytoplasmic DNA receptor cGAS is activated. Activated cGAS undergoes a conformational change and forms a dimer, which promotes the binding of ATP and GTP to generate cGAMP. cGAMP in turn binds to STING on the ER, which subsequently recruits TBK1. Phosphorylated TBK1 in turn activates IRF3. STING also activates IKK kinase, which phosphorylates the IκB family. The degradation of phosphorylated IκB proteins allows NF-κB to enter the nucleus and work with interferon regulatory factors such as IRF3 to induce the expression of interferons and inflammatory cytokines such as TNF, IL-1β and IL-6. Created by Figdraw. Export ID: RYIOTb98b8.

cGAS is a cytoplasmic DNA sensor that is present in an autoinhibited state when it is not bound to DNA (3). When exogenous or endogenous DNA is detected, cGAS binds to it in a DNA sequence-independent manner (4). Activated cGAS undergoes a conformational change to form a dimer, which is warranted for cGAS activation (5). Active cGAS dimers can promote the synthesis of ATP and GTP loops, secondary the second messenger known as cyclic GMP-AMP (cGAMP) (6). In turn, cGAMP binds to STING on the endoplasmic reticulum (ER) to form dimers, tetramers, and higher-order oligomers. Activated STING is transferred from the ER to the Golgi apparatus. During this process, STING recruits TANK-binding kinase 1 (TBK1). Phosphorylated TBK1, in turn, activates interferon regulatory factor 3 (IRF3). Dimerized IRF3 can trigger the production of type-I interferon (IFN) (7). In addition, STING activates IKK kinase, which phosphorylates the IκB family of inhibitors of nuclear factor-kappa B (NF-κB). The phosphorylated IκB protein is degraded via the ubiquitin-proteasome pathway; at this point, NF-κB enters the nucleus and functions together with IFN regulatory factors such as IRF3 to induce the expression of IFNs and inflammatory cytokines such as tumor necrosis factor (TNF), interleukin (IL)-1β, and IL-6 (3) (**Figure 1**).

## 3 Function of the cGAS-STING pathway in diseases

In recent years, researches on the cGAS-STING pathway have focused on the role of this pathway in various aspects such as tumor immunity, viral infection, inflammation, and aging.

The activation of the cGAS-STING pathway in cancer cells triggers the autocrine and paracrine secretion of type I IFN. The infiltration rate of immune cells and the expression profile of immune-related genes are increased (8), augmenting the activation and infiltration of CD8+ T cells (9, 10). Moreover, the antigenicity of cancer cells is increased, increasing the possibility of being recognized and killed by cytotoxic T cells; this results in tumor growth inhibition *in vivo* (11, 12). In addition, mutant P53 can inhibit the cGAS-STING pathway, leading to tumors escaping the immune system (13).

Besides, the double-stranded DNA from exogenous pathogens and mitochondrial DNA produced by damaged organisms can activate the cGAS-STING signaling pathway to induce a variety of inflammatory responses (14), leading to tissue fibrosis.

In atherosclerosis, diabetic cardiomyopathy, and myocardial infarction models, the cGAS-STING pathway is activated and downstream molecules are expressed. However, STING inhibitors can block fibrosis and apoptosis in cardiomyocytes, protecting myocardial function and delaying heart failure progression (14–17).

Activation of the cGAS-STING signaling pathway is also involved in the development of inflammatory injury and fibrosis in the liver, lungs, intestine, and central nervous system. On the other hand, inhibition of the cGAS-STING pathway decreases the occurrence of inflammation, and organ damage and improves these pathologies (18–26).

Increasing evidence suggests that the cGAS-STING signaling pathway plays a key pathogenic role in autoimmune diseases such as systemic lupus erythematosus (SLE), rheumatoid arthritis (RA), Aicardi-Goutières syndrome (AGS), and STING-associated vasculopathy with onset in infancy (SAVI). It was found that the expression of the IFN-inducible gene IFIT3 and downstream IFNβ were increased in patients with SLE compared with healthy controls; furthermore, it was positively correlated with the activity of the cGAS-STING pathway (27). In addition, IFN-stimulated genes (ISGs) are abnormally elevated in patients with AGS (28).

In a mouse model of inflammatory arthritis, cGAS deficiency blocked the IFN response and decreased inflammatory cell infiltration and joint swelling (29). Deletion of cGAS or STING can saved DNase II-/- mice from fatal inflammatory diseases caused by DNA clearance defects (30) (**Table 1**).

**Table 1:** Regulators of the cGAS-STING pathway.

Diseases	Function	Ref.
Tumor	IFN-I secretion, Immune cell infiltration, Enhanced tumor antigen presentation	(8, 9, 11, 12)
Diabetic cardiomyopathy	Phosphorylation of downstream targets NF-κB and IRF3 increased in mice model	(14)
Arteriosclerotic plaques	STING expressed in plaques near cellular debris and hemorrhagic lesions	(17)
Myocardial Infarction	Induces apoptosis and fibrosis	(15)
Acute Liver Injury	Causes liver damage and liver fibrosis	(25)
Schistosomiasis japonica	cGAS deficiency decreased egg granulomas and liver fibrosis	(24)
Industrial pulmonary fibrosis	Promotes lung inflammation and fibrosis	(23)
Colitis	Increased STING expression is a feature of intestinal inflammation in mice with colitis and patients with inflammatory bowel disease	(22)
Neurodegenerative diseases	Induces neuroinflammation	(19–21, 26)
SLE	IFN-β、IFIT3 high expression is positively correlated with the activity of the cGAS-STING signaling pathway	(27)
Irritative arthritis	cGAS deficiency reduced inflammatory cell infiltration and joint swelling	(29)
Aicardi-Goutières syndrome	cGAS/STING is a key nucleic acid-sensing pathway relevant to AGS	(28)

The cGAS-STING signaling pathway inhibits a variety of viral infections. During viral infections, viral replication induces the responses of inflammatory factors and ISGs, and the cGAS-STING pathway is activated, subsequently inhibiting the progression of viral infection. However, deletion or silencing of cGAS or STING genes results in reduced production of type I IFN and enhanced viral infection, thus suggested that the host cGAS-STING signaling pathway plays an important role in limiting viral replication (31–34).

The recruitment, activation, and signaling of each molecule of the cGAS-STING pathway are inextricably linked to the role of this pathway. A study has observed that cGAS is not only present in the cytoplasm but also in the nucleus and that nuclear cGAS plays a role in innate immunity against viral infections by regulating histone arginine modification (35). The deubiquitination of STING promotes its stability as well as the expression of type I IFN and pro-inflammatory factors after DNA viral infections (36). Furthermore, ubiquitin-regulated X structural domain protein promotes the ubiquitination, dimerization, and transport of STING and positively regulates STING signaling and subsequent TBK1 recruitment and phosphorylation to promote antiviral immune responses (37). The recruitment of TBK1 to STING is important for both IRF3 and NF-κB activation; furthermore, the resulting type I IFN -mediated independent immune defense against viral infections is essential (38).

**Figure 2:**
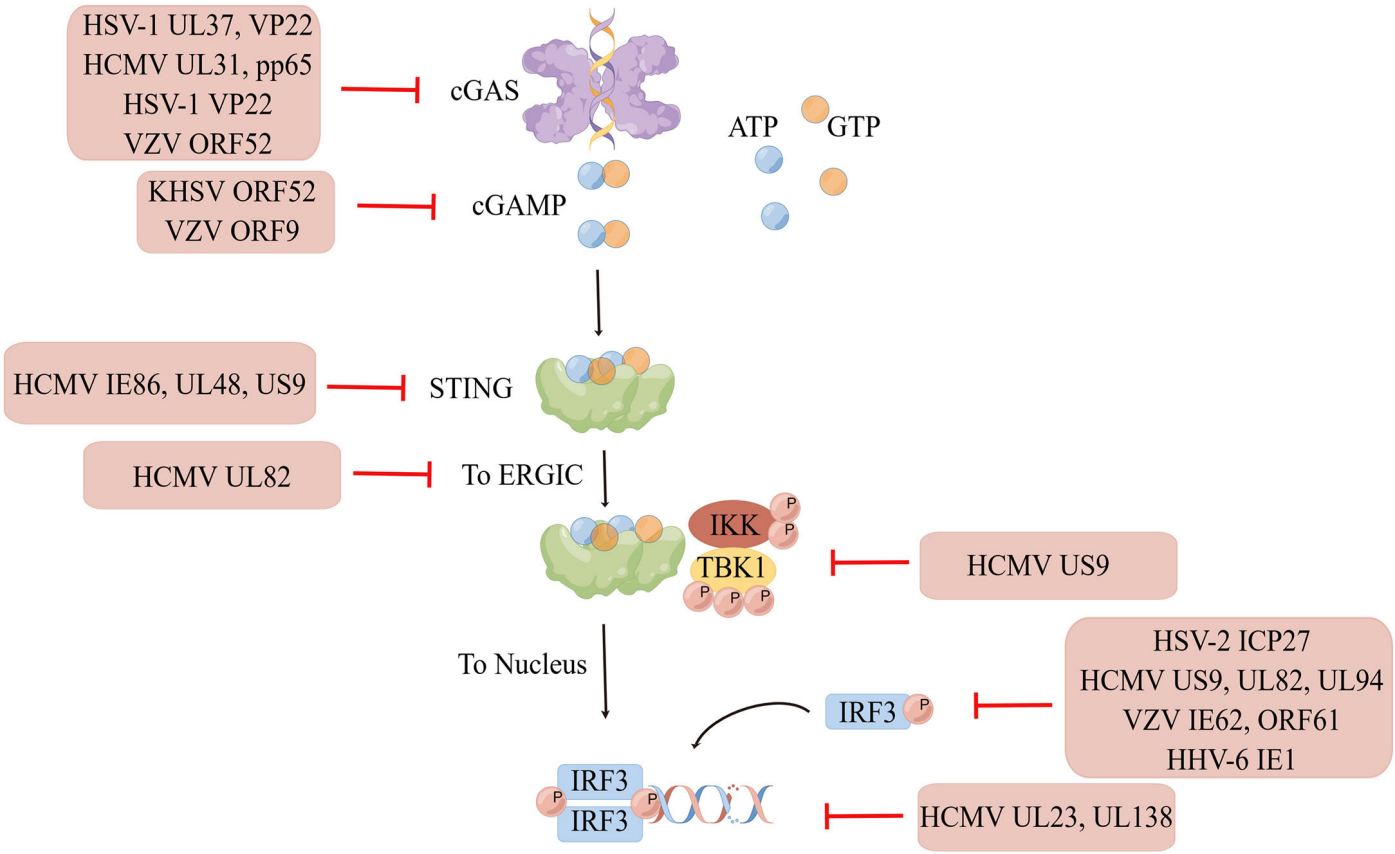
Immune escape of HHVs. Human herpesviruses can evade immune surveillance and maintain their replication by inhibiting the enzyme activity of cGAS, reducing the phosphorylation level of STING, TBK1, IRF3, and suppressing interferon gene activation. Created by Figdraw. Export ID: STPWPba3de.

The interaction between virus and host is exceptional because viral infection leads to the activation of the host cell’s natural immunity against the virus, which has evolved multiple strategies to escape the host’s antiviral immunity and establish latent infection. Various viral proteins can function as inhibitors of intracellular DNA receptors and antagonize the antiviral responses of the body. Furthermore, they can interact with various molecules of the cGAS-STING pathway, achieving immune escape by inhibiting the enzymatic activity of cGAS; reducing the phosphorylation levels of STING, TBK1, and IRF3; and inhibiting the activation of the IFN gene so as to maintain viral replication. Immune escape of the virus not only occurs during initial infection but also persists during the latent and recurrence periods of the virus, with the interaction between the strengthening of the body’s immune system and virus evolution constantly occurring (**Figure 2**).

## 4 Herpesvirus

Herpesviruses are a class of double-stranded DNA viruses that comprising a four-layered structure: an envelope, a tegument, a capsid, and a DNA core. They are one of the largest known viruses to date, with a diameter of approximately 200 nm. This virus class is divided into three subtypes: alpha, beta, and gamma, with highly prevalence worldwide. At present, nine types of herpesviruses can infect humans: herpes simplex virus-1 (HSV-1), herpes simplex virus-2(HSV-2),varicella-zoster virus(VZV), Epstein-Barr virus(EBV), human cytomegalovirus (HCMV), HHV-6A, HHV-6B, HHHV-7, and Kaposi’s sarcoma-associated herpesvirus(KSHV).Herpesvirus particles comprise a double-stranded DNA core and an icosahedral capsid surrounded by an unstructured proteinaceous matrix called the envelope. In turn, the envelope is surrounded by a lipid bilayer studded with branched glycoproteins (39).

HSV-1 primarily leads to cold sores, and neonatal herpes simplex encephalitis via vertical transmission from the mother to the child. On the other hand, HSV-2 primarily leads to genital herpes that manifests as clusters or scattered small blisters around the external genitalia or anus, which rupture after 2-4 days to form erosions or ulcers and are painful. After the initial genital herpes subsides, the remaining virus will be dormant for a long time; when the body’s resistance is decreased or provoking factors are encountered, the condition can relapse, with the recurrence symptoms being generally milder than the symptom of the initial infection.

Humans are the only natural host of VZV, and the skin is the main target organ of this virus. Varicella is more common among infants and preschool children and manifests as fever and patches of erythematous maculopapular rashes, herpes, and crusts on the skin and mucous membranes. The disease is self-limited and does not leave any scars; furthermore, lifelong immunity is achieved after illness. Herpes zoster is a common disease in adults, the elderly, or individuals with immunodeficiency and immunosuppression. It is caused by the activation of latent viruses. In the early stage, there is an abnormal feeling on the local skin such as itching or pain, followed by red rashes and herpes along the nerve distribution of the trunk.

EBV, also called HHV-4, is the causative agent of infectious mononucleosis and is strongly associated with the development of Burkitt lymphoma, nasopharyngeal cancer, and childhood lymphoma.

The clinical manifestations include pharyngitis, fever, and enlarged lymph nodes. HCMV, also called HHV-5, mostly exists in the form of latent infection. The general population is susceptible to HCMV, and can be repeatedly infected. Most HCMV infections are asymptomatic; however, HCMV can cause visceral diseases in some patients as well as infectious mononucleosis. Furthermore, HCMV causes neonatal infection via vertical transmission from the mother to the child, and congenital HCMV infections can be teratogenic in newborns. HHV-6 is more likely to infect patients with immunodeficiency. The clinical features of HHV-6 infection in adults are acute fever, upper respiratory tract symptoms, mononucleosis and rash. The rash is mostly in the form of macules, can be fused into pieces, and even develop into diffuse erythema; when the infection subsides, desquamation is observed.

On the other hand, HHV-6 infection in neonates is generally asymptomatic Only in severe cases, it may result in an infantile rash. In addition, HHV-7 infection is common in children. It can cause exanthema subitum, febrile seizures, or neurological complications. KSHV also called HHV-8 is closely related to the occurrence of Kaposi’s sarcoma in patients with AIDS.

Classic Kaposi’s sarcoma is characterized by purple, reddish-blue, or dark brown maculopapular rashes, plaques, and nodules, with ulcer formation in some cases. It is particularly common at the extremities and may be accompanied by lymphedema. HHV can be latent in the ganglia for a long time after the initial infection and can relapse when human immunity is weakened. To date, no effective vaccines against HHV have been developed (**Table 2**).

**Table 2:** Human herpesvirus and associated diseases.

HHV	Associated Diseases
HSV-1	Herpes of the mouth, lips and throat Neonatal herpes encephalitis
HSV-2	Genital Herpes
VZV	Chickenpox, Herpes zoster
EBV	Infectious mononucleosis, Burkitt lymphoma, nasopharyngeal cancer, and childhood lymphoma
HCMV	Infectious mononucleosis, Neonatal malformation
HHV-6	Mononucleosis, exanthema subitum
HHV-7	Exanthema subitum
KSHV	Kaposi’s sarcoma, AIDS

## 5 cGAS-STING pathway and HHV infections

### 5.1 cGAS-STING pathway and HSV infections

HSV-1 infection triggers the cGAS-STING and Toll-like receptor 3 pathways. Both these pathways are essential to attenuate viral replication (40). Some researchers have hypothesized that in HSV-1 infection, STING binds to NLR family pyrin domain containing 3 *via* two pathways to promote inflammasome activation and that the cGAS-STING-NLRP3 signaling pathway is crucial for the host to resist HSV-1 infection (41). When the cGAS-STING pathway is inhibited because of various reason or when type I IFN signaling is impaired, the innate antiviral immune response is also inhibited; as a result, HSV replication is enhanced, and the host is more resistant to this virus (42–44). Compared with adults, neonates with HSV infection, neonatal cord blood mononuclear cells and peripheral blood mononuclear cells displayed significantly decreased cGAS expression at the mRNA and protein levels. Furthermore, the production of cGAMP, a secondary messenger, and activation of the transcription factor IRF3 were markedly decreased, possibly leading to the high susceptibility of neonates to DNA viral infections (45).

HSV-1 can induce cytokine responses and apoptosis (46). Studies on clinical cases of herpes simplex encephalitis, mouse models, and primary cell cultures have reported that when microglia are infected with HSV-1, a low viral load can induce type I IFN response, whereas a high viral load can induce cGAS-STING pathway-dependent apoptosis (47). Moreover, in a mouse model of genital herpes, specific activation of the STING pathway in the vagina causes activation of the IFN system, limiting inflammatory responses to control HSV-2 infection *in vivo (* 48).

HSV is a DNA virus that has developed multiple strategies to evade host immune responses. Some HSV proteins can act on cGAS to achieve immune escape. For example, the HSV-1 surface protein UL37 can deamidate cGAS in human and mice. Deamidation impairs the ability of cGAS to catalyze cGAMP synthesis. HSV-1 with a deamidase deficiency in UL37 induces a potent antiviral response (49). Moreover, VP22, an HSV-1 body tegument protein, can interact with cGAS to inhibit its enzymatic activity and help continuously evade the innate antiviral response of the host (50). In addition, VP22 can effectively disrupt pre-formed cGAS DNA cohesion in cells, and ORF9, a tegument protein of VZV, acts similarly to inhibit cGAS-STING phase separation (51).

VP1-2, a deubiquitinating enzyme (DUB) of HSV-1 can act on STING, TBK1, IRF3 and other pathway molecules. DUB can decrease the phosphorylation levels of TBK1 and IRF3 by inhibiting the ubiquitination of STING, thereby realizing the immune escape of HSV in the brain. Human or mouse microglia infected with HSV1with a DUB-active mutant of VP1-2 protein can increase the expression of IFNs and decrease viral replication in the brain (52). Moreover, ICP27, an immediate-early protein of HSV-2, can directly bind to IRF3 and inhibit its phosphorylation and nuclear translocation (53).

The surface proteins of HSV-1 are effective IFN antagonists. For example, HSV-1 US3 functions against the antiviral immunity of the host by targeting the activation of β-catenin in the cGAS-STING-pathway (54). Furthermore, VP22 can downregulate IFN-γ promoter activation and IFN-γ production as well as inhibit the expression of IFN and its downstream antiviral genes (50). Taken together, VP22 plays a role in inhibiting the cGAS - STING-mediated antiviral innate immune signaling pathway. In addition, HSV-2 immediate early protein ICP27 inhibited the activation of IFN-β promoter and the production of IFN-βat the mRNA and protein levels (53). HSV-2 glycoprotein E and glycoprotein C act synergistically to protect the virus from antibody- and complement-mediated neutralization (55).

### 5.2 cGAS-STING pathway and HCMV infections

STING et al. PRRs pathway can recognize CMV and generate type I IFN-based immune defense to resist infection (56). In CMV-infected cell and animal models, STING is needed to initiate first-stage type I IFN production and inhibit CMV replication (57). Studies have reported that primary human endothelial cells produce a strong type I IFN response to HCMV invasion; this depends on cGAS, STING, and IRF3 signaling. Furthermore, HCMV can stimulate primary human monocyte-derived macrophages and dendritic cells and trigger type I IFN production in a cGAS-dependent cGAMP formation way. In addition, IFN-γ-inducible protein 16(IFI16) recognizes the herpesvirus genome and induces inflammasome production and IFN-β responses. As a restriction factor of viral lytic replication, IFI16 inhibits viral DNA replication, particularly HCMV replication (58, 59). Studies have reported that monocyte-derived cells without cGAS exhibit an impaired type I IFN response (60, 61).

In addition, CMV can evade the antiviral immunity of the body at multiple stages. First, upstream of the pathway, HCMV UL31 protein, cGAS inhibitor, can directly interact with cGAS, inhibit the enzymatic function of cGAS, and decrease cGAMP production. Furthermore, the overexpression of UL31 can significantly decrease the antiviral responses, whereas UL31 knockdown can upregulate the expression of type I IFN and downstream antiviral genes. In addition, wild-type HCMVs can replicate more efficiently than UL31-deficient HCMVs (62). PP65, a tegument protein of HCMV selectively binds to cGAS and prevents its interaction with STING, inactivating signaling via the cGAS-STING-IRF3 axis and inhibiting IFN-β production (63). Infection with the PP65-deficient mutant virus can reduce stronger IFN responses and proinflammatory chemokines (64).

HCMV can interact with STING and its downstream molecules. For example, the IE86 protein of HCMV can promote the proteasome-dependent degradation of STING. Furthermore, UL122, encoding the IE86 protein, can strongly inhibit STING-induced IFN-β promoter activation (65). UL82, a tegument protein of HCMV, can function as a negative regulator of STING-dependent antiviral responses and inhibit the transport of STING from the ER to perinuclear microsomes. Furthermore, it can inhibit the recruitment of TBK1 and IRF3 to the STING complex. Wild-type HCMV exhibits better replication efficiency than UL82-deficient mutants (66). Moreover, pUL48, a tegument protein of HCMV, can encode DUB for the deubiquitination of TRAF6, TRAF3, IRAK1, IRF7 and STING, thereby inhibiting type I IFN responses, obtaining tumor precursor function, and promoting tumor formation (67). HCMV US9 can disrupt STING oligomerization and STING and tbk1 binding via competitive interactions. Furthermore, it can inhibit the nuclear translocation of IRF3 and its cytosolic domain to inhibit IRF3 activation (68).

PUL83, a tegument protein of HCMV, can interact with IFI16 to stimulate the immediate early promoter of the virus and inhibit IFN signaling, thereby inhibiting the expression of antiviral genes in infected cells and achieving immune escape (64, 69, 70). Ul23, a tegument protein of HCMV, can significantly decrease the expression of ISGs and the activity of the promoter of the response elements of ISGs during HCMV infection. UL23 is a key factor in the negative regulation of type I IFN-mediated immune responses (71). HCMV inhibits the cGAS-STING-TBK1 pathway and decreases IFN-β mRNA accumulation via its latent associated protein UL138 (72).

UL94, an epidermal protein of HCMV, inhibits cGAS-STING-mediated antiviral responses, interacts with IRF3-activated mediator STING, disrupts STING dimerization and translocation, and hinders the recruitment of TBK1 to the STING signalosome. Ectopic expression of UL94 can impair cytoplasmic double-stranded DNA and DNA virus-induced type I IFN induction and enhance viral replication. In contrast, UL94 deficiency can enhance HCMV-induced transcription of type I IFN and downstream antiviral effectors and impair viral replication (73).

### 5.3 cGAS-STING pathway and KSHV infections

A study have reported that mitochondrial DNA on the surface of the extracellular vesicles (EVs) of KSHV is it’s a causative factor (74). KSHV-infected cells induce ISG responses. ISGs and IRF-activating genes were significantly activated in the EVs of human endothelial cells treated with KSHV EVs. This suggests that the cGAS-STING pathway is associated with KSHV EV-mediated expression of ISGs (74). In addition, some researchers have suggested the importance of STING in limiting bystander cell transmission than in inhibiting viral replication when endotheliocytes are infected with KSHV (75). Study have reported that new KSHV and HSV-1 infections and latent KSHV and EBV infections can induce the interaction of the H2B-IFI16-BRCA1 complex with intracellular cGAS and STING, leading to the phosphorylation of TBK1 and IRF3, nuclear translocation of IRF3, and production of IFN-γ (76).

The cGAS-dependent response induced by KSHV infection can limit infection, whereas KSHV also exhibits unique mechanisms to antagonize host cGAS sensing (77). ORF52 is a tegument protein abundantly present in extracellular viral particles. Studies have shown that KSHV cGAS inhibitors (KicGAS) encoded by *ORF52* inhibit DNA-induced phase separation and cGAS activation, thereby inhibiting cGAS enzymatic activity. KicGAS optimizes the production of infectious virus particles in addition to immune evasion. The loss of ORF52 reduces virion production and causes infectious defects in the virus, with the concomitant enhancement of cGAS signaling (77–79). Viral IFN regulatory factor 1(vIRF1), inhibits STING phosphorylation and activation by preventing the STING and TBK1interaction, thus inhibiting the DNA-sensing pathway. Virf1-expressing cells can inhibit IFN-β production after infection with pathogenic DNA. In summary, gamma herpesviruses encode inhibitors to block cGAS-STING-mediated antiviral immunity. The regulation of this pathway is important for the transmission and lifelong persistence of herpesviruses in the population (80).

The cGAS-STING pathway is activated during KSHV primary infection, as well as plays an important role in reactivating KSHV latency. Latency-associated nuclear antigen (LANA) in KSHV is mainly localized in the nucleus of latently infected cells and is essential for maintaining and replicating of latently infected viral DNA. The cytosolic isoforms of LANA act as antagonists of the cytosolic DNA sensor cGAS. LANA and its KSHV isoform inhibit TBK1 and IRF3 phosphorylation and cGAS-STING dependent IFN production by directly interacting with cGAS during virus latency reactivation. This eventually antagonizes the restriction of KSHV replication and counteracts innate immune responses, thus promoting virus reactivation in cells (81, 82).

### 5.4 cGAS-STING pathway and VZV infections

Type I IFN induction during VZV infection depends on the cGAS/STING DNA sensing pathway, and VZV recognition by cGAS limits its replication (83). STING mediated the host defense of dermal cells against VZV infection. STING inhibition by small interfering RNA- or short hairpin RNA-mediated gene disruption increased viral replication, reduced IRF3 phosphorylation, and induced IFN and proinflammatory cytokines. The pretreatment with STING agonists reduced VZV glycoprotein E levels and viral replication. Moreover, increased IFN-λ secretion in the STING-dependent pathway was observed after VZV infection (84). A study showed that transcripts of STING-encoding genes were selectively concentrated in exosomes secreted by VZV-infected lymphocytes (85). Reduced STING expression increased viral replication in primary fibroblasts, whereas STING overexpression inhibited VZV plaque formation (86).

Some VZV proteins are powerful antagonists of the IFN signaling pathway. The VZV immediate early proteinORF61 eliminates the natural immune response by degrading activated IRF3 and downregulating the IRF3-mediated IFN-β pathway (87). In addition, the VZV immediate early protein IE62 induces VZV gene expression upon VZV entry into cells and inhibits IFN-dependent antiviral defense. IE62 blocks TBK1-mediated IFN-β secretion and IRF3 phosphorylation (88). The VZV tegument protein ORF9 inhibits cGAMP production by functioning as a cGAS antagonist. Virus-expressed ORF9 binds to endogenous cGAS, resulting in an attenuated response of type I IFN (83).

### 5.5 cGAS-STING pathway and other HHVs

The levels of STING and programmed cell death ligand 1 (PD-L1) were significantly higher in EBV-associated gastric cancer than in non-EBV-associated gastric cancer. STING levels in EBV-associated gastric cancer were positively correlated with PD-L1 levels (89). C-176, a STING inhibitor, inhibits the EBV-induced transformation of peripheral blood mononuclear cells. Furthermore, C-176 treatment inhibited tumor formation and prolonged survival in a mouse model of EBV-associated lymphoid tissue proliferative disease (90). In human airway epithelial cells, HHV type IV EBV induces tripartite motif-containing protein 29 to suppress innate immune activation, leading to persistent DNA viral infection (91). The immediate early protein IE1 of human HHV type 6 is one of the first viral proteins synthesized upon virus entry and is a potent inhibitor of IFN-β gene expression. A study showed that in the presence of IE1, IRF3 did not efficiently bind to the IFN-β promoter sequence, and the dimerization and nuclear translocation of IRF3 decreased in the IE1-expressing cells (92). Study has found that the STING/STAT6 pathway was upregulated in HHV-6A-infected natural killer (NK) cells. NK cells infected with HHV-6B and HHV-7 showed significantly increased chemokine C-C motif ligand (CCL) 3, IFN-α, TNF-α, IL-8, and IFN-γ levels and slightly increased IL-4 and CCL4levels. HHV-6A-infected NK cells showed significantly increased IL-4 and IL-13 levels and slightly increased IL-10, TNF-α, IFN-α, and IFN-γ levels (93) (**Table 3**).

**Table 3:** Immune escape of HHVs.

Virus	Protein	Approach	Ref.
HSV-1	UL37	Deacetylated cGAS	(49)
	VP22	Inhibited enzymatic activity of cGAS	(50)
		Disrupted cGAS-DNA agglomeration	(51)
HSV-2	ICP27	Inhibited phosphorylation and nuclear shift of IRF3	(53)
	gE2, gC2	Protected the virus from antibody and complement neutralization synergistically	(55)
HCMV	UL31	Inhibited enzymatic activity of cGAS	(62)
	pp65	Block the interaction of cGAS and STING	(63)
	IE86	Promoted the degradation of proteasome-dependent STING	(65)
	UL82	Inhibited the transport of STING from ER to perinuclear microsomes, Hindered recruitment of TBK1 and IRF3	(66)
	UL48	Deubiquitinated STING	(67)
	US9	Disrupted the oligomerization of STING and combination of STING and TBK1, Blocked the nuclear transposition of IRF3	(68)
	UL83	Interacts with IFI16 to inhibit IFN signaling	(64, 69, 70)
	UL23	Reduced interferon stimulus response elements promoter activity	(71)
	UL138	Reduced the accumulation of IFN-β mRNA	(72)
	UL94	Interacted with IRF3-activated mediator STING, disrupted the dimerization and translocation of STING	(73)
KHSV	ORF52	Inhibits cGAMP produced by cGAS	(77–79)
VZV	ORF9	Inhibited cGAMP produced by cGAS	(83)
	ORF52	Disrupted cGAS-DNA agglomeration	(51)
	ORF61	Degraded activated IRF3	(87)
	IE62	Blocked the phosphorylation of IRF3	(88)
HHV-6	IE1	Inhibited dimerization and nuclear translocation of IRF3	(92)

## 6 Modulators of the cGAS-STING pathway

Various disease models, including autoimmune diseases, inflammation, organ damage and fibrosis, tumor occurrence and treatment, and viral infection, have been used in studies on modulators related to the cGAS-STING pathway. With the help of computer simulations or high-throughput sequencing, various compounds that act on this pathway have been artificially synthesize based on the spatial structure of cGAS, STING, or their ligands.

Modulators of the cGAS-STING pathway were first developed for tumor therapy, and STING agonists have been used in combination with other drugs to inhibit tumor growth. For example, the combination of cGAMP, a STING activator, with saponin adjuvants can increase the effectiveness of influenza vaccines in elderly hosts without additional doses or additional vaccinations (94). The combination of cGAMP and celecoxib, the cyclooxygenase 2 inhibitor, induces local and systemic antitumor immunity, thereby inhibiting tumor growth (95). The combination of STING agonists and eribulin, which is used to treat triple-negative breast cancer, increased the levels of the immunogenic cytokine IFNβ (96). The combination of STING agonists and atezolizumab inhibits breast cancer 4T1 cell growth in mic by increasing the levels of TNF-α, IFN-β, IL-10, and IFN-γ in surrounding blood and tumor masses (P < 0.01). In a tumor-bearing mouse model, these two drugs increased CD8+ cytotoxic T cells and reduced FOXP3+ T regulatory cells (97).

The systemic administration of STING agonists raises some safety concerns, and intratumoral injections are limited by tumor accessibility. Some researchers have combined STING agonists with antibodies targeting tumor cells via a cleavage linker to obtain antibody-drug conjugates (ADCs). ADCs exert strong anti-tumor effects and show good tolerance for systemic administration. STING ADCs promote multiple aspects of innate and adaptive anti-tumor immune responses, including the activation of dendritic cells, T cells, NK cells, NK T cells and the promotion of M2 to M1 polarization of tumor-associated macrophages (98). Some researchers have combined STING agonists with capsid-like hollow polymer nanoparticles. These nanoparticles are morphologically similar to natural virions and can facilitate co-presentation of antigens and STING agonists to increase immune responses (99).

Similarly, to overcome limitations associated with drug delivery in the body, researchers have designed nanoparticles for intratumoral injection. These nanoparticles are endowed with deoxyribonuclease resistance, which increases cellular uptake and promotes the escape of IFN-stimulated DNA endosomal into the cytoplasm, eventually mediating the production of pro-inflammatory cytokines by strongly activating the STING pathway of cGAS (100). The binding of STING agonists to these nanoparticles reduced reduce tumor burden by > 50% - 80% and significantly increased median survival in melanoma (YUMM1.7) and malignant breast adenoma (E0771) models (101). In the B16-f10 mouse melanoma lung metastasis model, lipid nanoparticle therapy containing a STING agonist improved anti-PD-1 resistance in the mice (102). Mn2+ is a cGAS-STING agonist that can significantly enhance anti-tumor immunity. Researchers introduced MnOx into the surface of Mn^2+^-loaded Pb nanoparticles to construct Mn^2+^ -rich photonic nanodrugs (MnPB-MnOx). All components of MnPB-MnOx were biocompatible and biodegradable, making cGAS-STING more available for activation. The local treatment of mice carrying 4T1 breast cancer cells *in situ* showed that MnPB-MnOx elicited a systemic response to suppress distal tumors (103).

STING agonists play an important role as antivirals. A synthetic analog of 3’,3’-c-di(2’F,2’dAMP), 2’,3’-cyclic GMP-AMP, and its precursor pivaloyloxymethyl exert strong anti-hepatitis B virus (HBV) effects in primary human hepatocytes regardless of HBV genotype (104). Zhang X et al. reported that the human STING (h-STING) agonist 6-bromo-N-(naphthalen-1-yl) benzo[d][1,3]dioxole-5-carboxamide can induce a broad-spectrum initial antiviral immunity. The treatment of primary human fibroblasts with this agonist-induced an antiviral state that inhibited several flavivirus infections (105). Naturally, STING can be activated by circular dinucleotides (CDNs) such as cGAMP. A synthetic CDN, ADU-S100, has been reported to be effective in activating STING and is being evaluated for the treatment of cancer in clinical trials. ADU-S100 is mostly injected intratumorally in animal experiments. The intraperitoneal injection of STING agonists can inhibit abnormal angiogenesis of tumors and can increase pericyte coverage. ADU-S100can normalize tumor vascularity and induce the formation of tertiary lymphoid structure in a tumor microenvironment (106), thereby promoting activated CD8+ T-cell infiltrate peritoneal tumor nodules (107).

The STING agonist 5,6-dimethylfuranone-4-acetic acid (DMXAA) was originally developed by the Auckland Cancer Society Research Centre as an anti-cancer drug and later discovered to be a mouse STING(m-STING) molecular-specific agonist. *In vivo* studies have shown that DMXAA can reduce the size of tumor blood vessels and increase the levels of tumor antigens (108). By performing single-cell RNA sequencing, researchers demonstrated that DMXAA could generate a chemokine environment to promote the recruitment of chimeric antigen receptor (CAR) T cells, thereby promoting the transport and persistence of CAR T cells (109). The antiviral action of DMXAA is crucial; DMXAA induces the STING signaling activation of macrophages in chronic HBV mice and inhibits the transcription of covalently closed circular DNA of HBV and its replication by epigenetic modification of hepatocytes (110). In addition, DMXAA administration to HSV-1-infected mice can reduce the viral load of their peripheral and central nervous systems, thereby increasing mouse survival (111). The cGAS-STING pathway promotes the development of inflammatory diseases, and DMXAA aggravates the severity of these diseases. A study showed that chronic exposure to DMXAA led to hepatic steatosis and inflammation in wild-type mice, but not in STING-deficient mice (112). In an acute pancreatitis model, DMXAA administration produced more severe symptoms in test mice than in control mice. Activated STING could sense acinar cell death by detecting DNA in acinar cells, and thus activated signaling pathways that promote inflammation (113). In cecal ligation perforation-induced sepsis mice, mice treated with the STING agonist DMXAA showed more intestinal cell apoptosis and severe systemic inflammatory responses than STING knockout mice (114).

Ramanjulu et al. developed a binding strategy to synergize the action of two symmetry-based amidobenzimidazole (ABZI) compounds. They developed ABZIs (diABZIs) with more powerful cell functions, strong binding capacity to STING (115), and more effectivity than that possessed by CDNs (116). diABZIs can stimulate h-STING molecules, induce type I IFN production, strengthen antiviral immunity, and exert inhibitory effects on various viral infections. Studies have shown that diABZIs exhibit potent antiviral activities against the human coronaviruses HCoV-229E and HCoV-OC43 via the IFN pathway and can prevent SARS-CoV-2 infection (117). Moreover, treatment with a low dose if diABZI (0.1 μM) effectively reduced the SARS-CoV-2 viral load on the surface of human small airway epithelial cells. After diABZI treatment, viral RNA levels measured by quantitative reverse transcription polymerase chain reaction were approximately 1000-fold (118). Furthermore, diABZI prevented epithelial damage in the reconstituted primary human bronchial airway epithelial acute lung injury (ALI) system (117). In k18-ace2 transgenic mice infected with SARS-CoV-2, the nasal administration of diABZI-4 before or after viral interference completely prevented the onset of severe respiratory diseases (119). The intravenous injection of diABZI into immunocompetent mice containing syngeneic colon tumors elicited strong antitumor activity and resulted in complete and durable tumor regression (115).

STING inhibitors can inhibit the activation of STING *in vivo*, thereby reducing organ damage and inflammatory responses.

Injecting the STING inhibitor C-176 in a diabetic cardiomyopathy mouse model significantly blocked inflammation and apoptosis in cardiomyocytes (14). C-176 administered mice effectively mitigated lung inflammation and fibrosis induced by graphitized multi-walled carbon nanotubes (23). Pretreatment with C-176 can prevent the nuclear displacement of NF-κB p65 and p-IRF3, thereby mitigating kidney damage in mice sensitized by trichloroethylene (120). The pharmacological blockade of STING with C-176 improves atherosclerotic formation in APOE-/- mice (121). Pretreating mice with acute lung injury with C-176 can reduce the production of inflammatory cytokines, including TNF-α, IL-6, IL-12, and IL-1β, and inhibit the gene expression of chemokines and adhesion molecule vascular cell adhesion protein-1 in lung tissues, thereby reducing lipopolysaccharide-induced mouse ALI (122). Researchers have developed spherical polyethyleneimine-coated mesoporous polydopamine nanoparticles loaded with C-176 (PEI-PDA@C-176 NPs) for RA treatment. The intra-articular injection of PEI-PDA@C-176 NPs effectively reduced dsDNA-induced arthritis and collagen-induced arthritis in a mouse model (123).

H-151, another novel STING inhibitor, targets STING molecules in humans and mice and protects various organs from damage. In a mouse model of intestinal ischemia-reperfusion(I/R) injury, H-151 treatment decreased p-IRF3, serum tissue damage marker (lactate dehydrogenase and aspartate aminotransferase), and cytokine (IL-1β and IL-6) levels, as well as reduced ischemia-reperfusion-induced intestinal and lung injuries and inflammation (124). In cisplatin-induced kidney injury mice, H151 treatment significantly improved the kidney injury; tubular cell apoptosis and tubular damage markers were effectively weakened, whereas renal function kidney morphology and renal inflammation were significantly improved. Cisplatin-induced mitochondrial damage and mitochondrial gene expression were reversed, mitochondrial morphology was improved, and mitochondrial DNA content was restored (125). In animal experiments, on the 28th day after myocardial infarction modeling in mice, the H-151 treatment exerted a significant inhibitory effect on the cGAS-STING-IRF3 pathway and inflammatory responses, especially the type I IFN response. Moreover, H-151 treatment reduced the apoptosis of adult cardiomyocytes and fibrosis of cardiac fibroblasts *in vivo*, thus protecting myocardial functions (15). Similarly, some researchers showed that three weeks after myocardial infarction in mice, theH-151 inhibited STING to reduce infarct dilation and scarring. Left ventricular systolic function increased to near-normal levels, and myocardial hypertrophy was reduced after H-151 treatment (16).

The cGAS inhibitor RU.521 attenuated the clinical signs of colitis in wild-type mice and decreased cGAMP and STING levels and TBK1 and IRF3 phosphorylation in colonic tissues (126). In pulmonary ventilation I/R rat models, cGAS-STING pathway inhibition attenuated ER stress, thereby reducing lung injuries and promoting lung functions (127). In a mouse model of sepsis, the cGAS inhibitor RU.521significantly increased cardiac functions and greatly reduced inflammatory responses, oxidative stress, and apoptosis in septic mouse hearts (128). In a mouse model of cerebral venous sinus thrombosis (CVST), RU.521 treatment reduced the levels of 2’3’-cGAMP, STING, and downstream inflammatory cytokines. It also reduced oxidative stress, decreased the number of microglia and neutrophils, improved neuronal apoptosis, and reduced neurological deficits caused by CVST (129).

In recent years, a series of compounds targeting the cGAS-STING pathway have been synthesized by different means.

Some of them are small molecule cyclic urea activators that can efficiently activate different STING molecules in humans (130), whereas some compounds are CDNs that can induce IFN secretion at higher levels (131). Furthermore, some of these compounds are thieno [2,3-d]imidazole derivatives that can induce tumor regression in mice without inducing weight loss as noval STING agonists (132), and some are oral STING agonists that can induce long-lasting antitumor immunity when combined with other drugs to overcome immunotherapy resistance synergistically (133). Moreover, some of these compounds are new covalent cGAS inhibitors that exert better inhibitory effects and selectivity than exerted by RU.521 (134), and some are new small-molecule human cGAS inhibitors with high binding affinity and cellular activity *in vitro* (135).

These approaches provide a basis for future studies to develop more efficient modulators for the cGAS-STING pathway and establish ideas for the clinical development of new therapeutic agents (**Table 4**).

**Table 4:** Regulators of the cGAS-STING pathway.

Regulators	Target	Name	Function	Ref.
Agonist	STING	cGAMP	Combined with saponin adjuvants to improve vaccine effectiveness	(94)
			Combined with COX-2 inhibitor celecoxib to inhibit tumor growth	(95)
			Combined with antineoplastic drug eribulin to enhance expression of IFN-β	(96)
			Combined with atezolizumab to inhibit breast cancer 4T1 cell growth in mice	(97)
		ADC	Promotes antitumor immune response, systemic administration is well tolerated	(98)
		Nanoparticle wrapped STING agonist	Promotes tumor antigen presentation	(99)
			Intratumoral injection enhances cell uptake	(100)
			Increases median survival in mice with melanoma (YUMM1.7) and breast malignant adenoma (E0771).	(101)
			Improves anti-PD-1 resistance in mice	(102)
		2’ F, 2’ dAMP, pivaloyloxymethyl	Anti-HBV	(104)
		BNBC	Induces broad-spectrum initial antiviral immunity	(105)
		ADU-S100	Promotes vascular normalization and the formation of tertiary lymphoid structures (TLS) within the tumor microenvironment	(106, 107)
		DMXAA	Reduces the size of tumor blood vessels *in vivo* and upregulates the expression of tumor antigens	(108)
			Promotes the transport and persistence of CAR T cells	(109)
			Inhibits hepatitis B virus replication	(110)
			Increased survival of HSV-1 infected mice	(111)
			Causes hepatic steatosis and inflammation in wild-type mice	(112)
			Exacerbates inflammation in mice with acute pancreatitis	(113)
			Exacerbates intestinal apoptosis and systemic inflammation in mice with sepsis	(114)
		diABZI	Complete and durable regression of colon tumors in mice	(115)
			Prevents epithelial injury in acute lung injury	(117)
			Block SARS-CoV-2 infection	(117–119)
Inhibitor	STING	C-176	Blocks inflammation and apoptosis of cardiomyocytes in diabetic DCM mice	(14)
			Reduces lung inflammation and fibrosis in mice	(23)
			Reduces kidney damage in mice caused by trichloroethylene sensitization	(120)
			Improve atherosclerotic formation in APOE-/- mice	(121)
			Mitigation of LPS-induced ALI in mice	(122)
		PEI-PDA@C-176 NPs	Mitigates of joint damage in mouse models of arthritis	(123)
		H-151	Reduces intestinal and lung injury in ischemia-reperfusion mice	(124)
			Improves cisplatin-induced kidney damage	(125)
			Reduce apoptosis of adult cardiomyocytes and fibrosis of cardiac fibroblasts, protects myocardial function	(15)
			Reduces myocardial infarction dilation and scarring, reduces myocardial hypertrophy	(16)
	cGAS	RU.521	Relieves wild-type mouse colitis	(126)
			Reduces lung injury in lung-ventilation-ischemia-reperfusion rats, promotes lung function	(127)
			Increases cardiac function in sepsis mice, reduces inflammatory response, oxidative stress and apoptosis in mouse hearts	(128)
			Reduces neurological deficits in CVST mice	(129)

## 7 Discussion

The summary of studies results in recent years showed that the cGAS-STING pathway played different roles in different disease models. We should look at its role dialectically.

In inflammatory and autoimmune diseases, the cGAS-STING pathway promotes inflammatory molecule secretion and recruitment, induces cell apoptosis, and aggravates the occurrence of fibrosis. However, in tumor development and antiviral infections, the cGAS-STING pathway plays a role in preventing disease development. It can promote CD8+ T-cell infiltration, change the tumor microenvironment, and inhibit tumor growth (97, 107). For antivirals, the activation of the cGAS-STING pathway increases the type-I-IFN-dominated antiviral responses (7).

HHV infection involves diverse susceptible people and disease spectra. HHV can cause neonatal infections, tumors, sexually transmitted diseases, and AIDS; thus, it is destructive to human health. Hence, attention should be paid to HHV infections and effective drugs should be promptly identified against the viral infections.

The cGAS-STING pathway exerts antiviral effects by inducing type I IFN production, providing a basis for treating HHV infection. However, HHVs also inhibit the cGAS-STING pathway via various viral proteins to escape immune surveillance. Viral proteins can interfere with various steps of the pathway, including inhibiting cGAS enzyme activity, reducing cGAMP secretion, disrupting cGAS-DNA binding, deubiquitinating STING, blocking STING intracellular transfer, inhibiting the recruitment of IRF3 and TBK1, inhibiting IRF3 phosphorylation and nuclear transport processes, and inhibiting IFN gene transcription. Thus, inhibiting viral replication may be possible by mutating these viral proteins. These findings provide insights for developing new antiviral agents.

In this review, we have summarized the role of the cGAS-STING pathway in different diseases, especially in anti- HHV infections, to bridge the gap in the interaction mechanism between some HHVs and the cGAS-STING pathway. We hope that this review provides a basis for future antiviral research as well as provides information on new targets and mechanisms to develop clinical drugs and vaccines.

However, the application of the cGAS STING pathway in diseases involves many aspects, and the discussion in the present review may not be detailed enough. EBV, HHV-6, HHV-7, and HHV-8 cause asymptomatic infections in the early clinical stage, and in some patients, symptoms only appear when the infections lead to serious complications or comorbidities. Thus, some gaps in the interaction between these viruses and the cGAS-sting pathway, which needs to be addressed and explored in future studies.

In recent years, agonists or inhibitors for the cGAS-STING pathway have also been actively developed. Small-molecule modulators with high efficacy and absorption rates and low side effects need to be developed. cGAS-STING pathway modulators have been studied intensively from the perspective of anti-tumor therapy. Many researchers have loaded STING agonists with nanoparticle, either by intratumoral injection or in combination with other drugs, to overcome limitations such as antitumor drug resistance and drug transportation in the body.

Besides, antiviral studies have shown that cGAS-STING pathway modulators exert good effects *in vivo* and *in vitro*; however, a few drugs are supported by clinical trial data (118, 136, 137). Many modulators have played an essential role theoretically *in vitro* and *in vivo*; however, owing to the specificity of human and mouse STING, as well as the metabolism and absorption of unknown drugs in the human body, cGAS-STING pathway modulators that can be used in clinical practice are yet to be explored. Therefore, future studies should be directed toward exploring such modulators.

## Author contributions

XJ and CH put forward the idea of this study. XJ prepared the initial manuscript, figures, and tables. XJ, CH, and ZC contributed to revising the final version. All authors contributed to the article and approved the submitted version.
